# Post-Translational Modifications of p53 in Ferroptosis: Novel Pharmacological Targets for Cancer Therapy

**DOI:** 10.3389/fphar.2022.908772

**Published:** 2022-05-24

**Authors:** Le Zhang, Ningning Hou, Bing Chen, Chengxia Kan, Fang Han, Jingwen Zhang, Xiaodong Sun

**Affiliations:** ^1^ Department of Endocrinology and Metabolism, Affiliated Hospital of Weifang Medical University, Weifang, China; ^2^ Clinical Research Center, Affiliated Hospital of Weifang Medical University, Weifang, China; ^3^ Department of Pathology, Affiliated Hospital of Weifang Medical University, Weifang, China

**Keywords:** ferroptosis, cancer, pharmacology, post-translational modifications, p53, cancer therapy

## Abstract

The tumor suppressor p53 is a well-known cellular guardian of genomic integrity that blocks cell cycle progression or induces apoptosis upon exposure to cellular stresses. However, it is unclear how the remaining activities of p53 are regulated after the abrogation of these routine activities. Ferroptosis is a form of iron- and lipid-peroxide-mediated cell death; it is particularly important in p53-mediated carcinogenesis and corresponding cancer prevention. Post-translational modifications have clear impacts on the tumor suppressor function of p53. Here, we review the roles of post-translational modifications in p53-mediated ferroptosis, which promotes the elimination of tumor cells. A thorough understanding of the p53 functional network will be extremely useful in future strategies to identify pharmacological targets for cancer therapy.

## 1 Introduction

The tumor suppressor protein p53 is often regarded as the gatekeeper of the cell because it is essential for stabilizing the cellular genome (Levine, 1997; Blagih et al., 2020). p53 exerts a tumor-suppressive function when exposed to DNA damage and various endogenous/exogenous stresses (Pietsch et al., 2008). Routinely activated p53 regulates cell cycle arrest and induces apoptosis to suppress cancer growth (Kastenhuber and Lowe, 2017). In contrast, 50% of human cancer cases are caused by biallelic mutations or deletions in the human gene *TP53*, leading to inappropriate activity of wild-type p53 and unrestrained tumor progression (Olivier et al., 2002). Thus, the preservation of p53 function to induce cell death is considered essential for cancer therapy.

In the past, apoptosis was regarded as the primary cell death mechanism in conventional cancer treatments. However, multidrug insensitivity or acquired resistance to existing traditional chemotherapy remains a major challenge for oncology treatment. Ferroptosis is an iron-dependent cell death process, characterized by excessive lipid peroxidation and iron overload (Dixon et al., 2012). Recently, ferroptosis has demonstrated considerable advantages in cancer treatment. Cancer cells are sensitive to ferroptosis because of their vigorous division and robust oxidative metabolic activity (Badgley et al., 2020). Additionally, cancer cells contain many targets for ferroptosis induction (Datta et al., 2007). Furthermore, ferroptosis can be combined with classical cancer treatments to reduce the survival and progression of malignant cells (Matsushita et al., 2015; Lin et al., 2016; Xu et al., 2018). For example, the anti-tumor medicine cisplatin can induce ferroptosis and apoptosis in lung cancer. Simultaneous application of cisplatin and erastin synergistically induces ferroptosis in colon and lung cancer cells (Guo et al., 2018). Thus, cancer cells with resistance to conventional chemotherapy, as well as cancer cells with a high propensity for metastasis, may be particularly vulnerable to ferroptosis.

Recently, the role of p53 biology in ferroptosis has received increasing interest for novel cancer treatments (Jiang et al., 2015). p53 is closely associated with key metabolic processes involved in ferroptosis (Liu and Gu, 2021). For example, p53 promotes cell survival by inhibiting ferroptosis in the absence of cysteine, suggesting an association between p53 and cysteine metabolism in ferroptosis (Tarangelo et al., 2018). Furthermore, ferroptosis is associated with both cancer and non-cancer diseases. Therefore, the regulation of ferroptosis by treatments that target p53 is a novel approach to cancer therapy.

Among the known regulatory processes, reversible post-translational modifications (PTMs) are regarded as critical regulators that control cellular quiescence and proliferation (Chen et al., 2020). PTMs are the most complex and efficient patterns that dynamically regulate the stability, conformation, and functions of p53 (Liu et al., 2019). Under various conditions, p53 can induce or suppress ferroptosis through transcriptional regulation or PTMs (Pietsch et al., 2008; Kang et al., 2019). However, the primary functions of PTMs of p53 in tumorigenesis-related ferroptosis have not been thoroughly reviewed in published literature. This review focuses on recent advancements in understanding the various PTMs of p53, as well as their roles in ferroptosis; we expect that this thorough analysis will promote research into the implications of those PTMs in cancer treatment.

## 2 Molecular Mechanisms of p53 in Ferroptosis-Based Cancer Therapy

**FIGURE 1:**
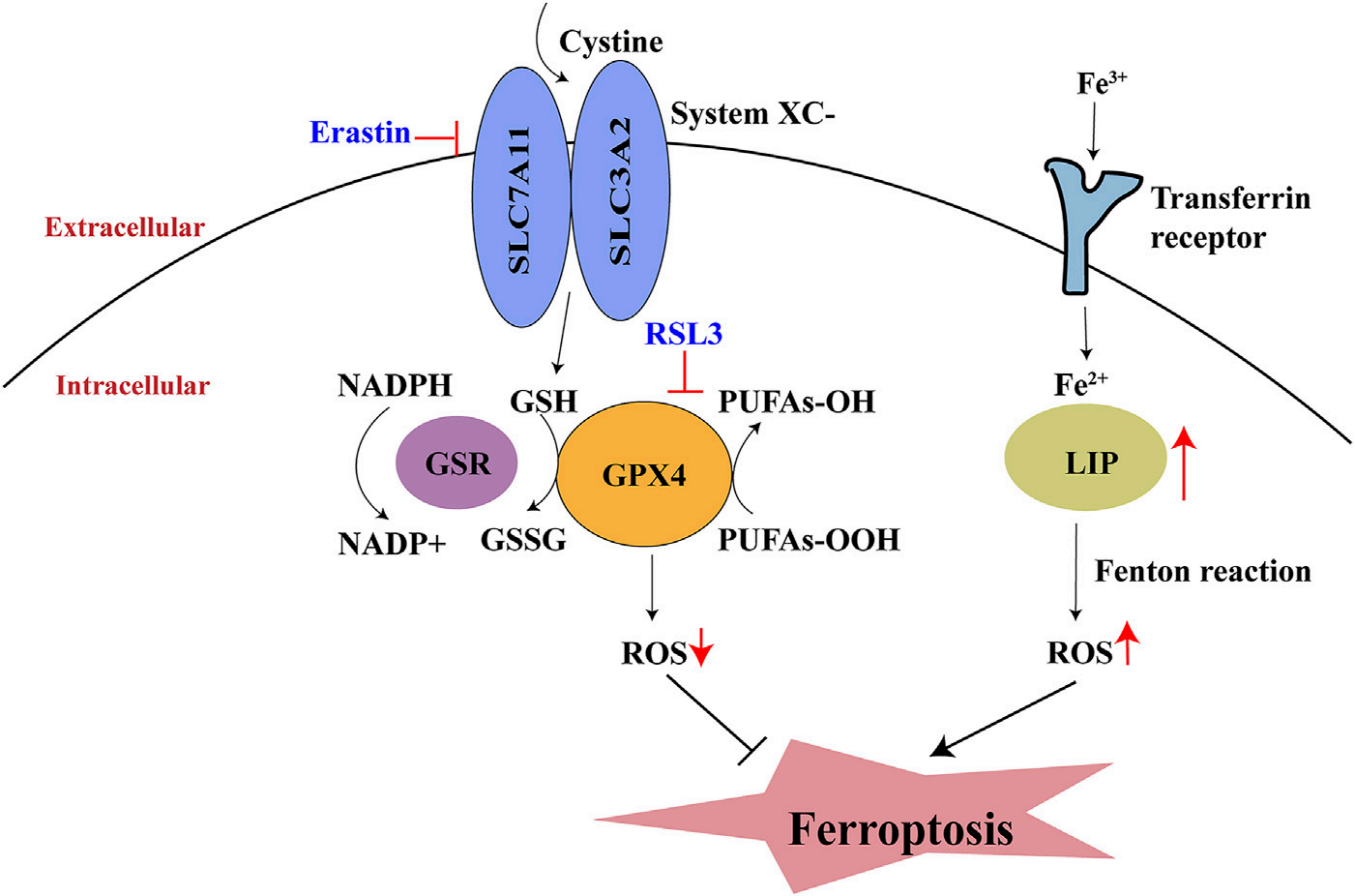
Process of ferroptosis. System Xc-, cystine/glutamate antiporter system; GSH, glutathione; GPX4, glutathione peroxidase; GSSG, oxidative glutathione; LIP, labile iron pool; NADPH, Nicotinamide adenine dinucleotide phosphate; PUFA, polyunsaturated fatty acid.

The term “ferroptosis” was initially used in 2012 by Dixon et al. (2012) to explain a new type of erastin-induced cell death that lacked features of apoptosis, necroptosis, and autophagy. The progression of ferroptosis is characterized by iron-dependent lipid peroxide injury in mitochondria; this injury involves inhibition of the cysteine/glutamate antiporter system (system Xc-), insufficient synthesis of glutathione (GSH), and depletion of glutathione peroxidase (GPX4) (Hassannia et al., 2019). The two key events in ferroptosis are dysregulation of lipid peroxidation and disruption of iron metabolism. Initially, cysteine is absorbed into the cytoplasm *via* system Xc-transporters; it then promotes GSH synthesis. GSH is then converted into oxidized glutathione (GSSG) *via* GPX4, thus reducing the amount of lipid-based reactive oxygen species (ROS) (Stockwell et al., 2017). Transferrin receptors import extracellular Fe^3+^ into cells; this Fe^3+^ is then converted to Fe^2+^ and transferred to the labile iron pool (Gkouvatsos et al., 2012). An increase in the labile iron pool leads to the accumulation of lipid ROS through the Fenton reaction; this eventually results in ferroptosis (Su et al., 2020) (Figure 1).

In 2015, Jiang et al. first identified a link between p53 and ferroptosis, such that p53 sensitized cells to ferroptosis (Jiang et al., 2015). Thus far, there have been numerous reports of p53-mediated ferroptosis. Generally, p53 is considered to be closely associated with cell cycle arrest, apoptosis, and senescence (Brady et al., 2011; Li et al., 2012; Valente et al., 2013). Usually, these processes are induced by p53 to repair DNA damage and prevent the development of malignant cells. Nonetheless, apoptosis evasion and enhanced resistance to apoptosis can impede cancer treatment (Guo et al., 2018). The discovery of p53 involvement in ferroptosis solved this problem. There have been multiple reports of combined cancer treatments that use conventional approaches and p53-mediated ferroptosis. For example, Lei et al. showed that radiotherapy-induced ferroptosis was also influenced by p53, leading to radiosensitivity in cancer cells. Some drugs for other diseases also have positive effects on p53-mediated ferroptosis. Sinapine, an alkaloid with antioxidant and anti-inflammatory properties, upregulates transferrin receptors in a p53-dependent manner that leads to ferroptosis in non-small cell lung cancer (Shao et al., 2022).

In addition to the p53-mediated ferroptosis network, several other networks mediate the induction of ferroptosis; these include autophagy proteins 5 and 7, the nuclear receptor coactivator four pathway, nuclear factor erythroid 2-related factor (Nrf2) in iron metabolism, and acyl-CoA synthetase long-chain family member four in lipid metabolism (Capelletti et al., 2020). However, these p53-independent types of ferroptosis have some limitations. For example, while enhanced expression of Nrf2 can reduce ROS levels and decrease stress responses (Suzuki et al., 2013), the activation of Nrf2 minimizes cancer cell sensitivity to conventional cancer treatments (e.g., chemotherapy and radiation) (DeNicola et al., 2011). This reduced sensitivity limits the usefulness of Nrf2-mediated ferroptosis in cancer therapy.

**FIGURE 2:**
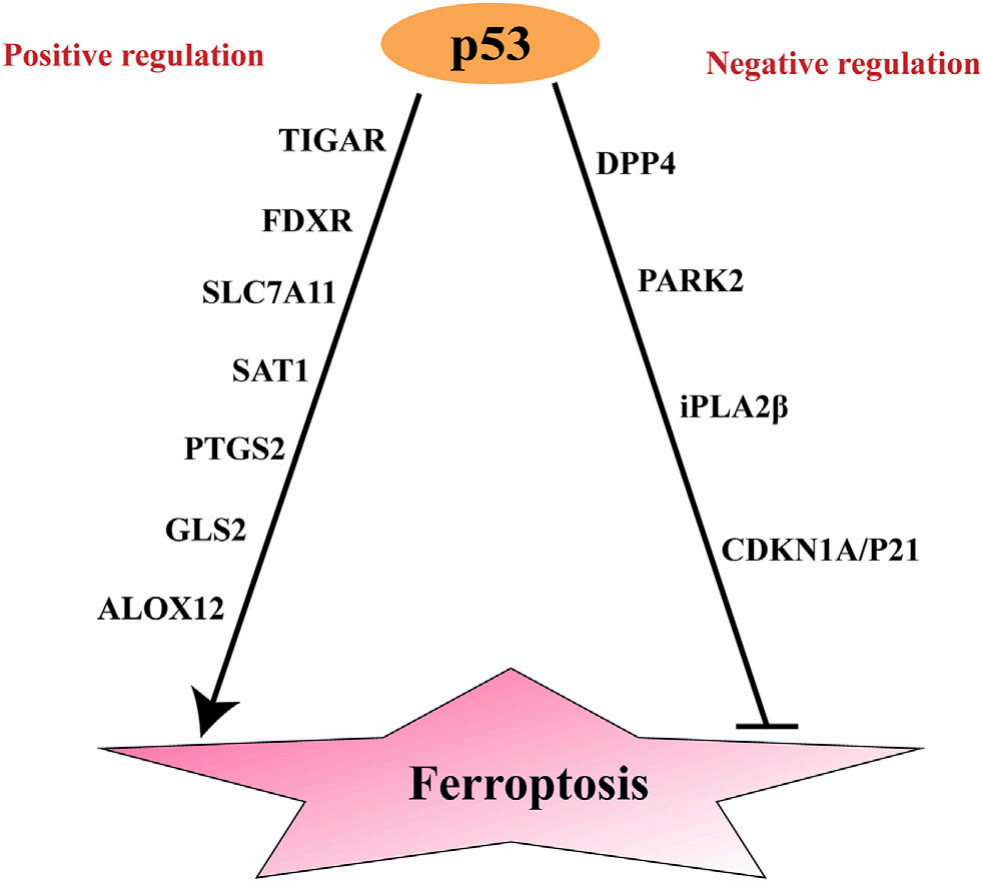
Positive and negative regulation of p53 to ferroptosis. SAT1, Spermidine/spermine N1-acetyltransferase; ; ALOX15, Arachidonic acid 15 lipoxygenases; GLS2, Glutaminases; PTGS2, Prostaglandin-endoperoxide synthase 2; ALOX12, Arachidonic acid 12 lipoxygenases; TIGAR, TP53-induced glycolysis and apoptosis regulator; FDXR, Ferredoxin reductase; DPP4, dipeptidyl peptidase-4; CDKN1A, encoding p21: cyclin-dependent kinase inhibitor; PARK2, parkinson disease 2.

Notably, p53 has two opposing effects on ferroptosis: it promotes and suppresses ferroptosis under specific conditions (Kang et al., 2019) (Figure 2). Under normal conditions, p53 can increase tumor cell sensitivity to ferroptosis and thus promote cell death. However, upon exposure to stresses such as cysteine deprivation, p53 delays ferroptosis (Friedmann Angeli et al., 2019). Differences in cell types and p53 mutation sites also influence the effects of p53 on ferroptosis in tumor cells. In the following sections, these opposing effects of p53 on ferroptosis will be clearly illustrated (Table 1).

**TABLE 1:** Regulation of p53 in ferroptosis network.

Downstream regulation of p53	Ferroptosis network alternations	Cancer type	Stress condition	Ways of regulation	References
SLC7A11	Regulate cystine metabolism	Human osteosarcoma U2OS cells	Nutlin-3 treatment; DNA damage	Positive regulation	Jiang et al. (2015)
					Saint-Germain et al. (2017)
					Yuan et al. (2022)
SAT1	Oxidize polyunsaturated fatty acids; increase lipid peroxidation	Melanoma cell line, A375	Nutlin and ROS treatment	Positive regulation	Ou et al. (2016)
					Saint-Germain et al. (2017)
GLS2	Reduce GSH; increase ROS	Human glioblastoma; HCC	Doxycycline existence; unstressed condition	Positive regulation	Hu et al. (2010)
					Wang et al. (2016)
					Jennis et al. (2016)
PTGS2	Regulate crucial membrane phospholipid; affect polyunsaturated fatty acids abundance and distribution	P53^3KR/3KR^ Mdm2^−/−^embryos	Not mentioned	Positive regulation	Jiang et al. (2015)
ALOX12	Activate lipoxygenase; induce ferroptosis	Human osteosarcoma cell	ROS stress	Positive regulation	Chu et al. (2019)
TIGAR	Maintain reduced state of GSH	Lung cancer cell	Adriamycin treatment	Positive regulation	Bensaad et al. (2006)
					Wang et al. (2016)
FDXR	Promote p53 expression; interact with p53; regulate iron metabolisms	Human colon cancer cells	Not mentioned	Positive regulation	Zhang et al. (2017)
PARK2	Eliminate damaged mitochondria; enhance GSH; reduce ROS	Lung cancer cells	Infrared radiation	Negative regulation	Zhang et al. (2011)
CDKN1A/p21	Consume intracellular GSH; reduce ROS level	Fibrosarcoma cells	Cystine deprivation; GPX4 inhibition	Negative regulation	Tarangelo et al. (2018)
iPLA2β	Downregulation of peroxidized membrane lipids	Melanoma cells	Nutlin; doxorubicin	Negative regulation	Chen et al. (2021)
DPP4	Diminish lipid peroxidation	Colorectal cancer	DPP4 inhibitor vildagliptin	Negative regulation	Xie et al. (2017)

ALOX12, Arachidonic acid 12 lipoxygenases; ALOX15, Arachidonic acid 15 lipoxygenases; CDKN1A/p21, Cyclin-dependent kinase inhibitor; DPP4, Dipeptidyl peptidase-4; FDXR, ferredoxin reductase; GLS2, glutaminases; PARK2, Parkinson disease 2; PTGS2, Prostaglandin-endoperoxide synthase 2; SAT1, Spermidine/spermine N1-acetyltransferase; TIGAR, TP53-induced glycolysis and apoptosis regulator.

### 2.1 Positive Regulation of Ferroptosis by p53

System X_C_- includes the transport component SLC7A11 and the regulatory module SLC3A2. SLC7A11 negatively regulates ferroptosis progression (Mou et al., 2019) and is overexpressed in various human cancers (Jiang et al., 2015; Koppula et al., 2021). In 2015, p53 was reported to promote ferroptosis through the transrepression of SLC7A11 expression (Jiang et al., 2015). Jiang et al. (2015) found that when p53 was activated by nutlin-3 or exposed to DNA damage stress, the expression of SLC7A11 was reduced. p53 binds to the promoter region of SLC7A11 to inhibit its expression, thus regulating cysteine metabolism and sensitizing cancer cells to ferroptosis.

Spermidine/spermine N1-acetyltransferase (SAT1) is a regulatory enzyme of the polyamine catabolism process (Pegg, 2008); it can be activated by oxidative stress, inflammation, and heat shock (Mandal et al., 2015). Because SAT1 is a transcriptional target of p53, SAT1 deficiency blocks p53-mediated ferroptosis in cancer. Upon exposure to combined treatment with nutlin and ROS, SAT1 induces p53-mediated ferroptosis (Ou et al., 2016). Although the p53–SAT1 axis increases cancer cell sensitivity to ferroptosis, this axis is not associated with GPX4-mediated ferroptosis (Ou et al., 2016). Notably, SAT1 overexpression leads to upregulation of arachidonic acid 15 lipoxygenase (ALOX15), which oxidizes polyunsaturated fatty acids and increases lipid peroxidation; these activities induce mitochondrial apoptosis and inhibit cell proliferation (Liu J et al., 2020). Taken together, these findings suggest that ALOX15 is a mediator of p53–SAT1 axis-induced ferroptosis in cancer (Ou et al., 2016).

Glutaminase 2 (GLS2) participates in glutamine metabolism; this enzyme is essential for the regulation of ferroptosis because it decreases glutathione and increases cellular ROS levels (Hu et al., 2010). GLS2 reportedly exhibited tumor-suppressive functions in liver and brain carcinomas with reduced levels of GSL2 (Hu et al., 2010; Liu et al., 2014; Martín-Rufián et al., 2014). The ability of p53 to bind to the GSL2 response element under stressed or unstressed conditions suggests that GSL2 can affect p53 function. GSL2 expression is responsible for p53-mediated oxygen consumption, mitochondrial respiration, and adenosine triphosphate production in cancer cells (Hu et al., 2010; Suzuki et al., 2010; Ou et al., 2016). However, further investigation is needed to determine whether GLS2 is critical for p53-mediated ferroptosis.

Prostaglandin-endoperoxide synthase 2 (PTGS2) encodes the enzyme cyclooxygenase 2, which catalyzes lipid oxidation (Liu J et al., 2020). PTGS2 regulates cellular sensitivity to ferroptosis by regulating phospholipid P, a critical membrane component. Both PTGS2 and SAT1 can affect the abundance and distribution of polyunsaturated fatty acids (essential substrates for ferroptosis), thus affecting lipid peroxidation and ferroptosis (Zheng and Conrad, 2020).

Arachidonic acid 12 lipoxygenase (ALOX12) is also closely associated with p53-mediated ferroptosis (Chu et al., 2019). ALOX12 inactivation abolishes p53-mediated ferroptosis in lymphoma. The potential mechanism is that, under ROS stress, p53 indirectly activates ALOX12 by transcriptional repression of SLC7A11 (Chu et al., 2019).

TP53-induced glycolysis and apoptosis regulator (TIGAR) is a target gene of p53 that inhibits cancer and has an antioxidant effect (Rajendran et al., 2013). Its possible role in ferroptosis is associated with its ability to maintain a lower level of cellular GSH. TIGAR promotes NADPH production through the pentose phosphate pathway, which has an important role in regulating subsequent NADPH-related ferroptosis (Ji et al., 2021).

p53 can also promote ferroptosis by influencing the function of ferredoxin reductase (FDXR) in iron metabolism (Ji et al., 2021). FDXR is a mitochondrial flavoprotein that transfers electrons to ferredoxin 1 (FDX1) and FDX2 (Brandt and Vickery, 1992); FDX1 is closely associated with steroidogenesis in mitochondria, while FDX2 is closely associated with cell survival. In the presence of FDX2 deficiency, mitochondrial iron overload occurs and p53 expression decreases, suggesting that FDX2 can transduce FDXR signals to regulate processes such as iron homeostasis, p53 expression, and tumor suppression (Zhang et al., 2017). p53 plays a role in iron homeostasis and mediates FDXR-dependent iron metabolism; the interaction between FDXR and p53 can also inhibit tumor initiation and progression. Notably, FDXR deficiency or overexpression combined with RSL3 and erastin led to the induction of ferroptosis, suggesting that FDXR is closely associated with the underlying induction mechanism. Additionally, FDXR-deficient mice are more likely to develop tumors (Zhang et al., 2017). These findings imply that the FDXR–p53 interaction inhibits tumorigenesis by maintaining iron homeostasis.

### 2.2 Negative Regulation of Ferroptosis by p53

In addition to its ferroptosis-promoting effects, p53 represses ferroptosis by inducing cyclin-dependent kinase inhibitor (CDKN1A, encoding p21), Parkinson disease 2 (PARK2) and iPLA2β, or by inhibiting dipeptidyl peptidase-4 (DPP4) (Kang et al., 2019) (Figure 2).

p53 targets CDKN1A/p21 in response to stress and senescence. p53-mediated CDKN1A/p21 expression hinders ferroptosis in response to cysteine deprivation (Tarangelo et al., 2018). In a recent study, p53-mediated CDKN1A expression delayed the onset of ferroptosis in response to subsequent cysteine deprivation in cancer cells (Tarangelo et al., 2018). This may be associated with reduced ROS production and attenuated GSH consumption (Ji et al., 2021). Importantly, that study was conducted in cells that had been pretreated with the p53 stabilizer nutlin-3; it remains unknown whether similar results can be achieved using other cells.

Furthermore, p53 targets PARK2 during mitophagy; PARK2 eliminates damaged mitochondria and attenuates sensitivity to ferroptosis in cancer (Zhang et al., 2011). Upon exposure to infrared radiation, p53-mediated expression of PARK2 is enhanced. p53 exerts its functions in mitochondrial respiration, oxygen consumption, and antioxidant defense by promoting PARK2 expression, suggesting that activation of the p53–PARK2 axis can limit cysteine deprivation-mediated ferroptosis. In PARK2-deficient mice, the tumor spectrum after exposure to infrared radiation was shorter than in wild-type mice, implying that PARK2 can serve as a tumor suppressor (Zhang et al., 2011). However, it is unclear whether p53 is involved in the inhibition of PARK2-mediated ferroptosis because p53 can also inhibit PARK2 activity (Hoshino et al., 2013; Gao et al., 2019).

In a recent paper, iPLA2β was reported to play a role in p53-mediated ferroptosis (Chen et al., 2021). Chen et al. (2021) found that iPLA2β could inhibit p53-mediated ferroptosis. When p53 was activated by nutlin or exposure to DNA damage, iPLA2β expression was increased. The loss of iPLA2β resulted in sensitivity to ferroptosis during ROS-induced stress in MCF7 cells and U2OS cells. This suppression mainly depended on the elimination of ALOX12-induced lipid peroxidation. In a xenograft mouse model, reduced expression of endogenous iPLA2β caused tumor cells to become sensitized to ferroptosis; thus, p53-mediated ferroptosis was enhanced. This finding suggested that iPLA2β could inhibit p53-driven ferroptosis.

The multifunctional protease DPP4 plays an essential role in cell death (Xie et al., 2017). In colorectal cancer, p53 regulates the cellular localization and activity of DPP4, rather than affecting its expression level (Xie et al., 2017). This effect is presumably because, in p53-deficient cells, DPP4 is located on the plasma membrane and binds to NADPH oxidase 1, thereby increasing lipid peroxidation and promoting ferroptosis. In colorectal cancer cells with high expression of DPP4, p53 binds and isolates DPP4 *via* ribozyme inactivation; this causes dissociation of NADPH oxidase one and nuclear translocation of DPP4, leading to diminished lipid peroxidation and abrogation of ferroptosis-promoting activity (Xie et al., 2017). However, this phenomenon has only been observed in colorectal cancer cells.

Thus, p53 is closely associated with the induction of ferroptosis. Regulation of p53 and its downstream genes (e.g., SLC7A11, iPLA2β, ALOX15, GLS2, TIGAR, PTGS2, FDR, DPP4, p21, and PARK2) may promote or inhibit the induction of ferroptosis. These are all known drug targets; however, new drugs can be designed for use in regulating ferroptosis and providing novel research insights.

## 3 Effects of p53 PTMs on Ferroptosis

### 3.1 p53 Functional Domains, Stability, and Activity

**FIGURE 3:**
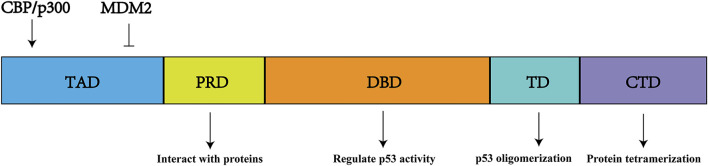
p53 structure and function. It includes six protein domains. TAD, N-terminal transactivation domains (s); DBD, central DNA-binding domain; PRD, proline-rich domain; TD, a tetramerization domain; CTD, C-terminal domain.

p53 is a type of sequence-specific DNA-binding protein that is referred to as a quantum jump (Bargonetti et al., 1991). The p53 protein contains 393 amino acids and six structural domains: two N-terminal transactivation domains, a central DNA-binding domain, a proline-rich domain, a tetramerization domain, and a C-terminal domain (Joerger and Fersht, 2016) (Figure 3). CBP/p300 (described in Section 3.2) and murine double minute-2 (MDM2), respectively, are positive and negative regulators that bind to sites on the N-terminal region of p53 (Mavinahalli et al., 2010). The DNA-binding domain allows p53 to bind to response elements on its target genes; thus, it is essential for p53 activity (Ryan, 2011). *TP53* mutations frequently occur in the DNA-binding domain, thereby disrupting the function of the p53 protein and its downstream network (Hainaut and Hollstein, 2000). Additionally, the proline-rich domain contains five PXXP motifs, which participate in interactions between p53 and various proteins; this domain is primarily responsible for the regulation of apoptosis (Lacroix et al., 2006). Finally, the tetramerization domain regulates the oligomerization of p53, while the C-terminal domain is associated with tetramerization of p53 (Golubovskaya and Cance, 2013).

Under normal physiological conditions, p53 is a housekeeping protein with a short half-life (Liu et al., 2019). However, in the presence of various types and levels of stress, p53 can be activated and accumulated to coordinate cellular responses. The activation of p53 is associated with cancer prevention; it initiates DNA repair, promotes apoptosis, influences ferroptosis, and regulates energy metabolism (Kaiser and Attardi, 2018; Zhao et al., 2021). Although multiple mechanisms have been proposed to explain the unique tumor suppression effects of p53, the specific function necessary for these effects remains unknown.

### 3.2 Post-Translational Modifications of p53 and Their Impacts on p53-Mediated Ferroptosis

**FIGURE 4:**
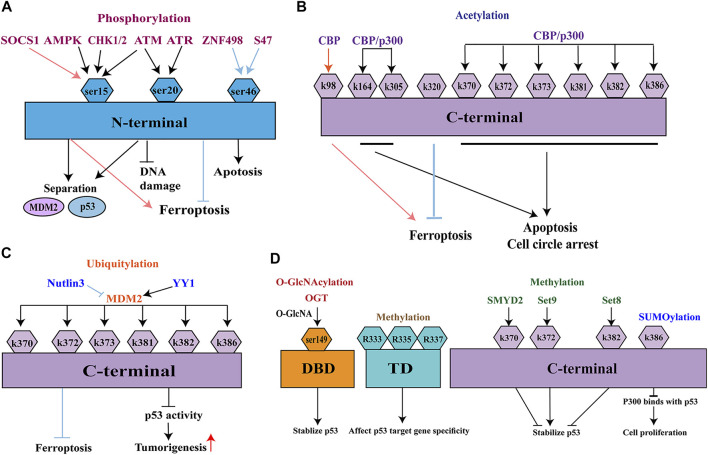
Post-translational modifications of p53. **(A)** phosphorylation; **(B)** acetylation; **(C)** ubiquitination; **(D)** O-GlcNAcylation, SUMOylation, and methylation. MDM2, Murine double minute-2; ATM, ataxia-telangiectasia mutated kinase; ATR, ATM-and Rad3-related kinase; CHK1/2, checkpoint kinase 1/2; AMPK, adenosine monophosphate-activated protein kinase; CBP, CREB binding protein; EP300/p300, E1A binding protein P300; PCAF, p300/CBP-associated factor; KAT5/Tip60, lysine acetyltransferase 5; KAT8/MOF, lysine acetyltransferase 8; MOZ, monocytic leukemia zinc finger; YY1, Yin Yang 1.

The paradigm shift concerning the role of p53 in ferroptosis prompted us to investigate how and when p53 promotes or suppresses ferroptosis. p53 activation is subject to a complex and diverse array of PTMs, which substantially influence the expression patterns of p53 target genes and related functional groups after the translation of p53 (Chen et al., 2020). PTMs of p53 can be rapidly reversed and constitute critical steps that greatly influence both carcinogenesis and cancer prevention (Gu and Zhu, 2012; Hassannia et al., 2019). PTMs of p53 mainly include phosphorylation, acetylation, ubiquitination, O-GlcNAcylation, SUMOylation, and methylation. This review focuses on the functional importance of the various PTMs in p53-mediated ferroptosis, specifically in the context of carcinogenesis and cancer prevention (Figure 4).

#### 3.2.1 Phosphorylation

Phosphorylation is the most widely studied protein modification (Figure 4A). p53 is usually phosphorylated at Ser15, which is located in a homologous subdomain of the N-terminal transactivation domain. Several kinases can phosphorylate p53 at these sites, including ataxia-telangiectasia mutated (ATM) kinase, ATM- and Rad3-related (ATR) kinase, and checkpoint kinase 1/2(CHK1/2) (Appella and Anderson, 2001). Because of their shared Ser20 phosphorylation mechanism, both ATM kinase and ATR kinase mediate the stabilization of human p53 in response to infrared- and ultraviolet-induced DNA damage (Chehab et al., 1999). Phosphorylation at Ser15 frequently occurs in glucose-dependent cell cycle arrest; it is regulated by adenosine monophosphate-activated protein kinase (AMPK) (Shieh et al., 1997; Jones et al., 2005; Yang et al., 2019). Phosphorylation at Ser15 results in a conformational change in the tertiary structure of p53; phosphorylation at Ser20 involves the tetramerization domain of p53. Both types of phosphorylation reduce the ability of p53 to bind its negative regulator MDM2, thereby improving p53 stability and function (Shieh et al., 1997; Shieh et al., 1999, Shieh et al., 2000; Teufel et al., 2009).

Suppressor of cytokine signaling 1 (SOCS1) is essential for the activation of p53-mediated ferroptosis and regulation of cellular senescence (Calabrese et al., 2009; Mallette et al., 2010). Saint-Germain et al. (2017) found that, under moderate DNA damage stress, SOCS1 phosphorylates Ser15 by promoting the interaction of p53 and ATM. Under high DNA damage stress, phosphorylation at Ser15 was independent of SOCS1, although SOCS1 continued to stabilize p53. SOCS1-mediated phosphorylation at Ser15 led to ferroptosis-related downregulation of SLC7A11 and upregulation of SAT1.

Among the known PTMs, Ser46 is another major phosphorylation site of the transactivation domain (Liebl and Hofmann, 2019). Phosphorylation of p53 at Ser46 has been implicated in p53 activation and its various pathophysiological effects. This type of phosphorylation induces apoptosis and ferroptosis in cells exposed to various stress (Zhang et al., 2022). An example is ZNF498, one of the Krüppel-associated box domain zinc-finger proteins. In ferroptosis-induced HepG2 cells, the overexpression of ZNF498 led to decreases in ROS production and GSL2 expression, as well as an increase in GSH production; these effects resulted in cell survival. Thus, overexpression of ZNF498 is presumably associated with tumor advancement and poor prognosis in hepatocellular carcinoma. A potential underlying mechanism involves attenuated phosphorylation of p53 at Ser46, which inhibits p53-mediated apoptosis and ferroptosis (Zhang et al., 2022). Another example is the Pro47Ser polymorphism (S47), which converts the proline residue adjacent to Ser46 in human p53 into serine (Lane, 2016). This change reduces ferroptosis by impairing p53-mediated downstream genes; it reduces apoptosis by decreasing the phosphorylation of Ser46 (Zhang et al., 2018). In a recent study, flubendazole (an anti-malarial drug) exhibited an anti-tumor effect by mediating the phosphorylation of p53; this promoted ferroptosis in castration-resistant prostate cancer. This process was associated with regulatory effects by SLC7A11 and GPX4 (Zhou et al., 2021). Further elucidation of the mechanisms that underlie p53 phosphorylation will yield new therapeutic strategies for ferroptosis-induced tumor suppression.

#### 3.2.2 Acetylation

Acetylation is also a common PTM that involves the modification of lysine residues in p53 (Figure 4B). Acetylation increases the stability of p53 and is essential for its ability to repair DNA damage (Kruse and Gu, 2009). Six p53 acetyltransferases have been identified, all of which modify p53 at lysines mainly in the C-terminus. These acetyltransferases include CREB-binding protein (CBP), E1A-binding protein P300 (EP300/p300), p300/CBP-associated factor (PCAF), lysine acetyltransferase 5 (KAT5/Tip60), lysine acetyltransferase 8 (KAT8/MOF), and monocytic leukemia zinc finger (MOZ). CBP/p300 is a transcriptional coactivator protein with acetyltransferase activity. Mutations in CBP/p300 commonly occur in several types of cancers, where they enhance histone acetylation and transcription of genes that surround the target gene (Goodman and Smolik, 2000; Luo et al., 2004). The transcriptional activity of p53 is enhanced by CBP/p300 through acetylation at C-terminal lysines (K370, K372, K373, K381, K382, K386, K164, and K305); these instances of acetylation lead to growth arrest and/or apoptosis (Dai and Gu, 2010). In a recent study, CBP acetylated residue K98 in mouse p53 (residue K101 in human p53) without disturbing p53 homeostasis, DNA-binding ability, or transcription activity (Wang et al., 2016). p53^3KR^ has a complete tumor suppression system (Brady et al., 2011). In a xenograft tumor model, a mutant p53^3KR^ (K117/161/162) that lacked acetylation capacity could not induce cell senescence and apoptosis; however, it repressed SLC7A11 expression and induced ferroptosis (Jiang et al., 2015). However, simultaneous mutation of K98 produced a new mutant (p53^4KR^), which lacked the ability to repress SLC7A11 expression and thus could not suppress tumor formation. Therefore, acetylation of p53 at K98 is essential for p53-mediated ferroptosis (Pegg, 2008; Lei et al., 2019). Mechanistic analysis indicated that combined removal of acetylation at K117/161/162 and K98 impeded p53-mediated transcriptional regulation of TIGAR and GLS2, which are closely associated with ferroptosis (Wang et al., 2016).

In addition to its effects on SLC7A11 suppression, p53 acetylation can regulate ferroptosis by regulating its downstream genes. Acetylation of p53 at K320 activates CDKN1A/p21, thereby delaying ferroptosis (Abbas and Dutta, 2009; Ji et al., 2021); however, the underlying mechanism is unknown. According to one hypothesis, p21 acts on GSH, thereby increasing the synthesis of GSH and GPX4, decreasing lipid peroxide accumulation, and reducing cell sensitivity to ferroptosis. However, the absence of cysteine leads to insufficient GPX4 synthesis, increased expression of wild-type p53, and increased cell sensitivity to ferroptosis (Bretscher et al., 2015). p53-mediated regulation of ferroptosis is closely associated with the levels of GPX4 and toxic peroxides. Therefore, p53 will affect cell sensitivity to ferroptosis by acting on p21 to control intracellular GSH levels, or by inhibiting SLC7A11 transcription (thereby increasing intracellular cysteine) (Ji et al., 2021). However, extensive studies regarding the mechanistic importance of p53 acetylation in tumor suppression are needed to determine the contributions of these acetyltransferases to the control of p53-mediated ferroptosis.

#### 3.2.3 Ubiquitination

Ubiquitination is a PTM of proteins that participates in the management of biological processes, immune responses, apoptosis, and cancer (Han et al., 2018) (Figure 4C). Ubiquitin-mediated proteasomal degradation tightly controls p53 activity at the cellular level. MDM2, a major E3 ubiquitin-protein ligase, is an oncoprotein overexpressed in many human cancers (Wade et al., 2013; Spiegelberg et al., 2018). It mainly targets six lysine residues in p53: K370, K372, K373, K381, K382, and K386 within the C-terminal domain (Rodriguez et al., 2000). Endogenous MDM2 is the major negative regulator of p53; it is highly specific for p53 ubiquitination. In the absence of stress, MDM2 maintains an appropriately low level of p53 by ubiquitinating the transactivation domain of p53 (Oliner et al., 1993; Zhu et al., 2018). Overexpression of MDM2 leads to inactive p53, resulting in the generation of infinitely replicating cells (Li et al., 2003). MDM2 interacts with the transcription factor Yin Yang1, which maintains binding between p53 and MDM2; this promotes p53 ubiquitination and represses p53 activity, thereby promoting tumorigenesis (Kruse and Gu, 2009). The relationship between p53 ubiquitination and ferroptosis differs according to cell type. Ferroptosis can be delayed by pretreatment with nutlin-3 (an inhibitor of MDM2), suggesting that stable and persistent p53 reduces the rate of ferroptosis (Ji et al., 2021). Nutlin-3-mediated activation of p53 significantly reduces the expression of SLC7A11 in HT-1080 human fibrosarcoma cells (Zhang et al., 2018). In addition to the above-mentioned roles, MDM2 and MDMX can induce ferroptosis in a p53-independent manner, which is associated with PPARα-mediated lipid regulation by the MDM2-MDMX complex (Venkatesh et al., 2020).

Additionally, MDM2-independent ubiquitination regulates p53 activity by influencing cell degradation and localization. A recent study reported dual regulatory effects of p62 on ferroptosis in glioblastoma (Yuan et al., 2022). In mutant p53 glioblastoma, p62 activates p53; it then promotes ferroptosis by inhibiting p53 ubiquitination and SLC7A11 expression. However, in p53 wild-type glioblastoma, p62 attenuates ferroptosis and promotes SLC7A11 expression.

Acetylation and ubiquitination are mutually exclusive because they both modify the same lysine residue in the C-terminus of p53. MDM2 binds to amino acids 17–28, while CBP/p300 binds to amino acids 22–26 (Golubovskaya and Cance, 2013). MDM2-mediated ubiquitination prevents p53 acetylation, thereby causing rapid proteasome-mediated degradation (Ito et al., 2001). Elucidation of the relationship between acetylation and ubiquitination could provide further insight into the biology of p53 and its tumor suppression effects.

#### 3.2.4 SUMOylation

SUMOylation, a reversible PTM of p53, has attracted increasing attention because it occurs in almost all eukaryotes (Figure 4D). SUMOylation participates in cellular death processes, maintains genome integrity, and regulates biological processes (Han et al., 2018). This process involves a small ubiquitin-like modifier (SUMO), which is a ubiquitin-like protein that is conjugated to lysines on p53 through a mechanism similar to ubiquitination. Thus far, five SUMO isoforms (SUMO1–5) have been identified in mammalian cells. Both SUMO1 and SUMO2/3 can SUMOylate p53 at K386 (Sheng et al., 2021). This SUMOylation prevents p300 from accessing C-terminal lysines and represses transcriptional activation, thus promoting cell proliferation by inhibiting the antigrowth function (Chen et al., 2020). Furthermore, SUMOylation can regulate the p53–MDM2 interaction, inhibiting tumor cell proliferation or inducing death (Carter et al., 2007).

SUMOylation is also implicated in ferroptosis (Stockwell et al., 2017) and is hyperactive in many cancers (Sheng et al., 2021). For example, Nrf2 can reduce ferroptosis by SUMOylation at K110 (Guo et al., 2019; Liu P et al., 2020). In addition, GPX4 can be SUMOylated at K125, which enables it to participate in ferroptosis (Sheng et al., 2021). However, few studies have explored the relationship between SUMOylation of p53 and ferroptosis; further investigations are needed.

#### 3.2.5 Methylation

Methylation is an important PTM of p53; it most commonly occurs on lysine and arginine (Figure 4D). It is an important epigenetic marker on lysines in histone tails, similar to acetylation. The first reported methylation of p53 was lysine methylation by Set9 methyltransferase at K372, which positively stabilizes p53 by stabilizing the chromatin-bound portion of p53 (Chuikov et al., 2004). p53 can also be methylated at K382 by Set8 or K370 by SMYD2, both of which negatively stabilize p53 (Huang et al., 2006; Shi et al., 2007). Thus, the activating or repressing effects of methylation on p53 function depend on the sites that are modified. p53 methylation may also occur at the arginine residues R333, R335, and R337; these affect p53 target gene specificity in response to DNA damage (Jansson et al., 2008). Methylation also interacts with other PTMs. Methylation at K372 suppresses inhibitory methylation at K370, while methylation at K382 competes with acetylation at the same site (Huang et al., 2006; Shi et al., 2007). A better understanding of the relationships among different PTMs under specific stress should provide important insights into the function of the p53 pathway in ferroptosis.

#### 3.2.6 O-GlcNAcylation

O-GlcNAcylation is another type of PTM, which involves the addition of N-acetylglucosamine (GlcNAc) to Ser or Thr residues (Figure 4D). O-GlcNAcylation is associated with cancer metabolism because of the Warburg effect in cancer cells, which exhibit high glycolytic flux and glucose uptake (de Queiroz et al., 2016). O-GlcNAcylation is mainly regulated by O-GlcNAc transferase and O-GlcNAcase, which are responsible for the addition or removal of O-GlcNAc. O-GlcNAcylation communicating with other PTMs may reveal the tumor-suppressive potential of the remaining p53 pathway. p53 can be O-GlcNAcylated at Ser149, thereby reducing proteasome-induced p53 degradation and stabilizing p53 (Yang et al., 2006). However, other O-GlcNAcylated sites have not been identified because Ser149 mutations do not lead to reduced levels of O-GlcNAcylation on p53. Furthermore, O-GlcNAcylation is reportedly associated with decreased phosphorylation of Thr155 at p53, which leads to increased protein stability, rather than interaction with MDM2 and subsequent ubiquitination (Yang et al., 2006). Acute exposure of endothelial cells to hyperglycemic conditions increases acetylation of p53 and expression of p21, suggesting that O-GlcNAcylation leads to increased transcription of p53 target genes (Zhang et al., 2015). Because O-GlcNAcylation has considerable effects in cellular metabolism, an emerging key question is whether the O-GlcNAcylation of p53 participates in the ferroptosis pathway.

### 3.3 Pharmacological Targets of p53-Mediated Ferroptosis Network for Cancer Therapy

Numerous studies have explored the pharmacological targets in p53-meditated ferroptosis for cancer therapy. SLC7A11 and GPX4 may be the most valuable pharmacological targets in the ferroptosis network. This has been confirmed by several studies on traditional antitumors drugs such as olaparib (Hong et al., 2021), tanshinone IIA (Guan et al., 2020) and bavachin (Luo et al., 2021). The poly (ADP-ribose) polymerase olaparib has been demonstrated to promote ferroptosis in ovarian cancer cells by downregulating SLC7A11 expression (Hong et al., 2021). This is a new way completely different from classical DNA repair function. Tanshinone IIA, the active ingredient of Chinese herbal medicine Salvia miltiorrhiza Bunge, also promotes ferroptosis in gastric cancer cells *via* p53/SLC7A11 (Guan et al., 2020). Bavachin induces ferroptosis in osteosarcoma cells *via* repressing SLC7A11 (Luo et al., 2021). Interestingly, bavachin also increases intracellular labile iron levels (Luo et al., 2021). This reveals that some drugs may not only act on a single target but promote ferroptosis through multiple pathways.

Targeting the p53/SLC7A11/GPX4 axis has also been found in non-anti-tumor drugs. Flubendazole, an antimalarial drug, inhibits SLC7A11/GPX4, promoting ferroptosis in castration-resistant prostate cancer (Zhou et al., 2021). Levobupivacaine, distinct from its anesthetic effect, inhibits SLC7A11/GPX4 and promotes p53-mediated ferroptosis to exert an antitumor effect in non-small cell lung cancer (Meng et al., 2021). Besides, other targets can also be utilized. For example, an increased dose of N-acylsphingosine amidohydrolase (ASAH2) inhibitor NC06 promotes p53 and heme oxygenase-1 and thus causes ferroptosis in colon cancer *via* decreasing oxidized glutathione (Zhu et al., 2021). The pharmacological targets described above bring new ideas for future research.

## 4 Targeting PTMs as a Perspective Strategy

Recently, a new generation of anticancer agents targeting PTMs has led to a revolutionary therapeutic approach that provides enhanced selectivity and context-specificity. Most anti-tumor small-molecule compounds are pan-inhibitors, which act on multiple proteins with similar modifications; thus, they cause unexpected side effects. p53 maintains a series of complex PTMs during its activation; for example, it exhibits different types of PTMs at specific lysine residues (acetylation, methylation, and ubiquitination). Thus, precision cancer treatment can be achieved by accurately targeting PTMs of p53 under specific conditions.

Several small-molecule inhibitors can block or promote the interplay between specific PTMs of p53 and corresponding upstream proteins; these inhibitors include aurora-A, ZNF498, and eupaformosanin (Hsueh et al., 2013; Wei et al., 2022; Zhang et al., 2022). For example, ZNF498 inhibits Ser46 phosphorylation to reduce p53 transcription in hepatocellular carcinoma (Zhang et al., 2022). This provides a novel perspective for the treatment of hepatocellular carcinoma by suppressing ZNF498. Eupaformosanin is a natural product that ubiquitinates p53 and can induce ferroptosis in triple-negative breast cancer (Wei et al., 2022). Additionally, some specific PTMs of p53 or its upstream proteins have been found play roles in ferroptosis. Ferroptosis can be regulated by CBP analogs or inhibitors such as C646 (Zhou et al., 2018). Among the various PTMs, acetylation of p53 might be the most powerful pharmacological target; however, this hypothesis requires further investigation.

## 5 Conclusion and Perspectives

Dysfunction of the tumor suppressor p53 has a widespread impact during carcinogenesis. The multiple functions of p53 and its numerous PTMs make its biology particularly complex. This review described diverse PTMs of p53 in cancer, with a focus on how p53 and its PTMs participate in ferroptosis. The dual effects (positive or negative) of p53 on ferroptosis are closely related to cancer cell type. PTMs are important components in the p53 signaling pathway; they strictly control the functional diversity of p53. Among the various PTMs of p53, acetylation has the greatest effects on p53-mediated ferroptosis during tumorigenesis. More detailed studies of how O-GlcNAcylation, SUMOylation, and methylation affect ferroptosis, as well as the corresponding networks, are needed to fully elucidate the landscape of p53 function in cancer treatment. Additionally, the biological effects of some drugs that target PTMs of p53 should be investigated to bring new insights to clinical cancer treatment.

## References

[B1] Abbas T. Dutta A. (2009). p21 in Cancer: Intricate Networks and Multiple Activities. Nat. Rev. Cancer 9, 400–414. 10.1038/nrc2657 19440234 PMC2722839

[B2] Appella E. Anderson C. W. (2001). Post-translational Modifications and Activation of P53 by Genotoxic Stresses. Eur. J. Biochem. 268, 2764–2772. 10.1046/j.1432-1327.2001.02225.x 11358490

[B3] Badgley M. A. Kremer D. M. Maurer H. C. DelGiorno K. E. Lee H. J. Purohit V. (2020). Cysteine Depletion Induces Pancreatic Tumor Ferroptosis in Mice. Science 368, 85–89. 10.1126/science.aaw9872 32241947 PMC7681911

[B4] Bargonetti J. Friedman P. N. Kern S. E. Vogelstein B. Prives C. (1991). Wild-type but Not Mutant P53 Immunopurified Proteins Bind to Sequences Adjacent to the SV40 Origin of Replication. Cell. 65, 1083–1091. 10.1016/0092-8674(91)90560-l 1646078

[B116] Bensaad K. Tsuruta A. Selak M. A. Vidal M. N. Nakano K. Bartrons R. (2006). TIGAR, A p53-Inducible Regulator of Glycolysis and Apoptosis. Cell 126, 107–120. 10.1016/j.cell.2006.05.036 16839880

[B5] Blagih J. Buck M. D. Vousden K. H. (2020). p53, Cancer and the Immune Response. J. Cell. Sci. 133, jcs237453. 10.1242/jcs.237453 32144194

[B6] Brady C. A. Jiang D. Mello S. S. Johnson T. M. Jarvis L. A. Kozak M. M. (2011). Distinct P53 Transcriptional Programs Dictate Acute DNA-Damage Responses and Tumor Suppression. Cell. 145, 571–583. 10.1016/j.cell.2011.03.035 21565614 PMC3259909

[B7] Brandt M. E. Vickery L. E. (1992). Expression and Characterization of Human Mitochondrial Ferredoxin Reductase in *Escherichia coli* . Arch. Biochem. Biophys. 294, 735–740. 10.1016/0003-9861(92)90749-m 1567230

[B8] Bretscher P. Egger J. Shamshiev A. Trötzmüller M. Köfeler H. Carreira E. M. (2015). Phospholipid Oxidation Generates Potent Anti-inflammatory Lipid Mediators that Mimic Structurally Related Pro-resolving Eicosanoids by Activating Nrf2. EMBO Mol. Med. 7, 593–607. 10.15252/emmm.201404702 25770125 PMC4492819

[B9] Calabrese V. Mallette F. A. Deschênes-Simard X. Ramanathan S. Gagnon J. Moores A. (2009). SOCS1 Links Cytokine Signaling to P53 and Senescence. Mol. Cell. 36, 754–767. 10.1016/j.molcel.2009.09.044 20005840

[B10] Capelletti M. M. Manceau H. Puy H. Peoc'h K. (2020). Ferroptosis in Liver Diseases: An Overview. Int. J. Mol. Sci. 21, 4908. 10.3390/ijms21144908 PMC7404091 32664576

[B11] Carter S. Bischof O. Dejean A. Vousden K. H. (2007). C-terminal Modifications Regulate MDM2 Dissociation and Nuclear Export of P53. Nat. Cell. Biol. 9, 428–435. 10.1038/ncb1562 17369817

[B12] Chehab N. H. Malikzay A. Stavridi E. S. Halazonetis T. D. (1999). Phosphorylation of Ser-20 Mediates Stabilization of Human P53 in Response to DNA Damage. Proc. Natl. Acad. Sci. U. S. A. 96, 13777–13782. 10.1073/pnas.96.24.13777 10570149 PMC24141

[B13] Chen D. Chu B. Yang X. Liu Z. Jin Y. Kon N. (2021). iPLA2β-mediated Lipid Detoxification Controls P53-Driven Ferroptosis Independent of GPX4. Nat. Commun. 12, 3644. 10.1038/s41467-021-23902-6 34131139 PMC8206155

[B14] Chen L. Liu S. Tao Y. (2020). Regulating Tumor Suppressor Genes: Post-translational Modifications. Signal Transduct. Target Ther. 5, 90. 10.1038/s41392-020-0196-9 32532965 PMC7293209

[B15] Chu B. Kon N. Chen D. Li T. Liu T. Jiang L. (2019). ALOX12 Is Required for P53-Mediated Tumour Suppression through a Distinct Ferroptosis Pathway. Nat. Cell. Biol. 21, 579–591. 10.1038/s41556-019-0305-6 30962574 PMC6624840

[B16] Chuikov S. Kurash J. K. Wilson J. R. Xiao B. Justin N. Ivanov G. S. (2004). Regulation of P53 Activity through Lysine Methylation. Nature 432, 353–360. 10.1038/nature03117 15525938

[B17] Dai C. Gu W. (2010). p53 Post-translational Modification: Deregulated in Tumorigenesis. Trends Mol. Med. 16, 528–536. 10.1016/j.molmed.2010.09.002 20932800 PMC2978905

[B18] Datta J. Majumder S. Kutay H. Motiwala T. Frankel W. Costa R. (2007). Metallothionein Expression Is Suppressed in Primary Human Hepatocellular Carcinomas and Is Mediated through Inactivation of CCAAT/enhancer Binding Protein Alpha by Phosphatidylinositol 3-kinase Signaling Cascade. Cancer Res. 67, 2736–2746. 10.1158/0008-5472.CAN-06-4433 17363595 PMC2276570

[B19] de Queiroz R. M. Madan R. Chien J. Dias W. B. Slawson C. (2016). Changes in O-Linked N-Acetylglucosamine (O-GlcNAc) Homeostasis Activate the P53 Pathway in Ovarian Cancer Cells. J. Biol. Chem. 291, 18897–18914. 10.1074/jbc.M116.734533 27402830 PMC5009264

[B20] DeNicola G. M. Karreth F. A. Humpton T. J. Gopinathan A. Wei C. Frese K. (2011). Oncogene-induced Nrf2 Transcription Promotes ROS Detoxification and Tumorigenesis. Nature 475, 106–109. 10.1038/nature10189 21734707 PMC3404470

[B21] Dixon S. J. Lemberg K. M. Lamprecht M. R. Skouta R. Zaitsev E. M. Gleason C. E. (2012). Ferroptosis: an Iron-dependent Form of Nonapoptotic Cell Death. Cell. 149, 1060–1072. 10.1016/j.cell.2012.03.042 22632970 PMC3367386

[B22] Friedmann Angeli J. P. Krysko D. V. Conrad M. (2019). Ferroptosis at the Crossroads of Cancer-Acquired Drug Resistance and Immune Evasion. Nat. Rev. Cancer 19, 405–414. 10.1038/s41568-019-0149-1 31101865

[B23] Gao M. Yi J. Zhu J. Minikes A. M. Monian P. Thompson C. B. (2019). Role of Mitochondria in Ferroptosis. Mol. Cell. 73, 354. 10.1016/j.molcel.2018.10.042 30581146 PMC6338496

[B24] Gkouvatsos K. Papanikolaou G. Pantopoulos K. (2012). Regulation of Iron Transport and the Role of Transferrin. Biochim. Biophys. Acta 1820, 188–202. 10.1016/j.bbagen.2011.10.013 22085723

[B25] Golubovskaya V. M. Cance W. G. (2013). Targeting the P53 Pathway. Surg. Oncol. Clin. N. Am. 22, 747–764. 10.1016/j.soc.2013.06.003 24012397 PMC3810242

[B26] Goodman R. H. Smolik S. (2000). CBP/p300 in Cell Growth, Transformation, and Development. Genes. Dev. 14, 1553–1577. 10.1101/gad.14.13.1553 10887150

[B27] Gu B. Zhu W. G. (2012). Surf the Post-translational Modification Network of P53 Regulation. Int. J. Biol. Sci. 8, 672–684. 10.7150/ijbs.4283 22606048 PMC3354625

[B28] Guan Z. Chen J. Li X. Dong N. (2020). Tanshinone IIA Induces Ferroptosis in Gastric Cancer Cells through P53-Mediated SLC7A11 Down-Regulation. Biosci. Rep. 40, BSR20201807. 10.1042/BSR20201807 32776119 PMC7953492

[B29] Guo H. Xu J. Zheng Q. He J. Zhou W. Wang K. (2019). NRF2 SUMOylation Promotes De Novo Serine Synthesis and Maintains HCC Tumorigenesis. Cancer Lett. 466, 39–48. 10.1016/j.canlet.2019.09.010 31546024

[B30] Guo J. Xu B. Han Q. Zhou H. Xia Y. Gong C. (2018). Ferroptosis: A Novel Anti-tumor Action for Cisplatin. Cancer Res. Treat. 50, 445–460. 10.4143/crt.2016.572 28494534 PMC5912137

[B31] Hainaut P. Hollstein M. (2000). p53 and Human Cancer: the First Ten Thousand Mutations. Adv. Cancer Res. 77, 81–137. 10.1016/s0065-230x(08)60785-x 10549356

[B32] Han Z. J. Feng Y. H. Gu B. H. Li Y. M. Chen H. (2018). The Post-translational Modification, SUMOylation, and Cancer (Review). Int. J. Oncol. 52, 1081–1094. 10.3892/ijo.2018.4280 29484374 PMC5843405

[B33] Hassannia B. Vandenabeele P. Vanden Berghe T. (2019). Targeting Ferroptosis to Iron Out Cancer. Cancer Cell. 35, 830–849. 10.1016/j.ccell.2019.04.002 31105042

[B34] Hong T. Lei G. Chen X. Li H. Zhang X. Wu N. (2021). PARP Inhibition Promotes Ferroptosis via Repressing SLC7A11 and Synergizes with Ferroptosis Inducers in BRCA-Proficient Ovarian Cancer. Redox Biol. 42, 101928. 10.1016/j.redox.2021.101928 33722571 PMC8113041

[B35] Hoshino A. Mita Y. Okawa Y. Ariyoshi M. Iwai-Kanai E. Ueyama T. (2013). Cytosolic P53 Inhibits Parkin-Mediated Mitophagy and Promotes Mitochondrial Dysfunction in the Mouse Heart. Nat. Commun. 4, 2308. 10.1038/ncomms3308 23917356

[B36] Hsueh K. W. Fu S. L. Chang C. B. Chang Y. L. Lin C. H. (2013). A Novel Aurora-A-mediated Phosphorylation of P53 Inhibits its Interaction with MDM2. Biochim. Biophys. Acta 1834, 508–515. 10.1016/j.bbapap.2012.11.005 23201157

[B37] Hu W. Zhang C. Wu R. Sun Y. Levine A. Feng Z. (2010). Glutaminase 2, a Novel P53 Target Gene Regulating Energy Metabolism and Antioxidant Function. Proc. Natl. Acad. Sci. U. S. A. 107, 7455–7460. 10.1073/pnas.1001006107 20378837 PMC2867677

[B38] Huang J. Perez-Burgos L. Placek B. J. Sengupta R. Richter M. Dorsey J. A. (2006). Repression of P53 Activity by Smyd2-Mediated Methylation. Nature 444, 629–632. 10.1038/nature05287 17108971

[B39] Ito A. Lai C. H. Zhao X. Saito S. Hamilton M. H. Appella E. (2001). p300/CBP-mediated P53 Acetylation Is Commonly Induced by P53-Activating Agents and Inhibited by MDM2. EMBO J. 20, 1331–1340. 10.1093/emboj/20.6.1331 11250899 PMC145533

[B40] Jansson M. Durant S. T. Cho E. C. Sheahan S. Edelmann M. Kessler B. (2008). Arginine Methylation Regulates the P53 Response. Nat. Cell. Biol. 10, 1431–1439. 10.1038/ncb1802 19011621

[B115] Jennis M. Kung C. P. Basu S. Budina-Kolomets A. Leu J. I. Khaku S. (2016). An African-Specific Polymorphism in the TP53 Gene Impairs p53 Tumor Suppressor Function in a Mouse Model. Genes Dev. 30, 918–930. 10.1101/gad.275891.115 27034505 PMC4840298

[B41] Ji H. Wang W. Li X. Han X. Zhang X. Wang J. (2022). p53: A Double-Edged Sword in Tumor Ferroptosis. Pharmacol. Res. 177, 106013. 10.1016/j.phrs.2021.106013 34856333

[B42] Jiang L. Kon N. Li T. Wang S. J. Su T. Hibshoosh H. (2015). Ferroptosis as a P53-Mediated Activity during Tumour Suppression. Nature 520, 57–62. 10.1038/nature14344 25799988 PMC4455927

[B43] Joerger A. C. Fersht A. R. (2016). The P53 Pathway: Origins, Inactivation in Cancer, and Emerging Therapeutic Approaches. Annu. Rev. Biochem. 85, 375–404. 10.1146/annurev-biochem-060815-014710 27145840

[B44] Jones R. G. Plas D. R. Kubek S. Buzzai M. Mu J. Xu Y. (2005). AMP-activated Protein Kinase Induces a P53-dependent Metabolic Checkpoint. Mol. Cell. 18, 283–293. 10.1016/j.molcel.2005.03.027 15866171

[B45] Kaiser A. M. Attardi L. D. (2018). Deconstructing Networks of P53-Mediated Tumor Suppression *In Vivo* . Cell. Death Differ. 25, 93–103. 10.1038/cdd.2017.171 29099489 PMC5729531

[B46] Kang R. Kroemer G. Tang D. (2019). The Tumor Suppressor Protein P53 and the Ferroptosis Network. Free Radic. Biol. Med. 133, 162–168. 10.1016/j.freeradbiomed.2018.05.074 29800655 PMC6251771

[B47] Kastenhuber E. R. Lowe S. W. (2017). Putting P53 in Context. Cell. 170, 1062–1078. 10.1016/j.cell.2017.08.028 28886379 PMC5743327

[B48] Koppula P. Zhuang L. Gan B. (2021). Cystine Transporter SLC7A11/xCT in Cancer: Ferroptosis, Nutrient Dependency, and Cancer Therapy. Protein Cell. 12, 599–620. 10.1007/s13238-020-00789-5 33000412 PMC8310547

[B49] Kruse J. P. Gu W. (2009). Modes of P53 Regulation. Cell. 137, 609–622. 10.1016/j.cell.2009.04.050 19450511 PMC3737742

[B50] Lacroix M. Toillon R. A. Leclercq G. (2006). p53 and Breast Cancer, an Update. Endocr. Relat. Cancer 13, 293–325. 10.1677/erc.1.01172 16728565

[B51] Lane D. (2016). p53: Out of Africa. Genes. Dev. 30, 876–877. 10.1101/gad.281733.116 27083994 PMC4840293

[B52] Lei P. Bai T. Sun Y. (2019). Mechanisms of Ferroptosis and Relations with Regulated Cell Death: A Review. Front. Physiol. 10, 139. 10.3389/fphys.2019.00139 30863316 PMC6399426

[B53] Levine A. J. (1997). p53, the Cellular Gatekeeper for Growth and Division. Cell. 88, 323–331. 10.1016/s0092-8674(00)81871-1 9039259

[B54] Li M. Brooks C. L. Wu-Baer F. Chen D. Baer R. Gu W. (2003). Mono- versus Polyubiquitination: Differential Control of P53 Fate by Mdm2. Science 302, 1972–1975. 10.1126/science.1091362 14671306

[B55] Li T. Kon N. Jiang L. Tan M. Ludwig T. Zhao Y. (2012). Tumor Suppression in the Absence of P53-Mediated Cell-Cycle Arrest, Apoptosis, and Senescence. Cell. 149, 1269–1283. 10.1016/j.cell.2012.04.026 22682249 PMC3688046

[B56] Liebl M. C. Hofmann T. G. (2019). Cell Fate Regulation upon DNA Damage: P53 Serine 46 Kinases Pave the Cell Death Road. Bioessays 41, e1900127. 10.1002/bies.201900127 31621101

[B57] Lin R. Zhang Z. Chen L. Zhou Y. Zou P. Feng C. (2016). Dihydroartemisinin (DHA) Induces Ferroptosis and Causes Cell Cycle Arrest in Head and Neck Carcinoma Cells. Cancer Lett. 381, 165–175. 10.1016/j.canlet.2016.07.033 27477901

[B58] Liu J. Zhang C. Wang J. Hu W. Feng Z. (2020). The Regulation of Ferroptosis by Tumor Suppressor P53 and its Pathway. Int. J. Mol. Sci. 21, 8387. 10.3390/ijms21218387 PMC7664917 33182266

[B59] Liu J. Zhang C. Lin M. Zhu W. Liang Y. Hong X. (2014). Glutaminase 2 Negatively Regulates the PI3K/AKT Signaling and Shows Tumor Suppression Activity in Human Hepatocellular Carcinoma. Oncotarget 5, 2635–2647. 10.18632/oncotarget.1862 24797434 PMC4058033

[B60] Liu P. Wu D. Duan J. Xiao H. Zhou Y. Zhao L. (2020). NRF2 Regulates the Sensitivity of Human NSCLC Cells to Cystine Deprivation-Induced Ferroptosis via FOCAD-FAK Signaling Pathway. Redox Biol. 37, 101702. 10.1016/j.redox.2020.101702 32898818 PMC7486457

[B61] Liu Y. Tavana O. Gu W. (2019). p53 Modifications: Exquisite Decorations of the Powerful Guardian. J. Mol. Cell. Biol. 11, 564–577. 10.1093/jmcb/mjz060 31282934 PMC6736412

[B62] Liu Y. Gu W. (2021). The Complexity of P53-Mediated Metabolic Regulation in Tumor Suppression. Seminars Cancer Biol. 10.1016/j.semcancer.2021.03.010 PMC8473587 33785447

[B63] Luo J. Li M. Tang Y. Laszkowska M. Roeder R. G. Gu W. (2004). Acetylation of P53 Augments its Site-specific DNA Binding Both *In Vitro* and *In Vivo* . Proc. Natl. Acad. Sci. U. S. A. 101, 2259–2264. 10.1073/pnas.0308762101 14982997 PMC356938

[B64] Luo Y. Gao X. Zou L. Lei M. Feng J. Hu Z. (2021). Bavachin Induces Ferroptosis through the STAT3/P53/SLC7A11 Axis in Osteosarcoma Cells. Oxid. Med. Cell. Longev. 2021, 1783485. 10.1155/2021/1783485 34707773 PMC8545544

[B65] Mallette F. A. Calabrese V. Ilangumaran S. Ferbeyre G. (2010). SOCS1, a Novel Interaction Partner of P53 Controlling Oncogene-Induced Senescence. Aging (Albany NY) 2, 445–452. 10.18632/aging.100163 20622265 PMC2933891

[B66] Mandal S. Mandal A. Park M. H. (2015). Depletion of the Polyamines Spermidine and Spermine by Overexpression of Spermidine/spermine N¹-acetyltransferase 1 (SAT1) Leads to Mitochondria-Mediated Apoptosis in Mammalian Cells. Biochem. J. 468, 435–447. 10.1042/BJ20150168 25849284 PMC4550555

[B67] Martín-Rufián M. Nascimento-Gomes R. Higuero A. Crisma A. R. Campos-Sandoval J. A. Gómez-García M. C. (2014). Both GLS Silencing and GLS2 Overexpression Synergize with Oxidative Stress against Proliferation of Glioma Cells. J. Mol. Med. Berl. 92, 277–290. 10.1007/s00109-013-1105-2 24276018 PMC4327995

[B68] Matsushita M. Freigang S. Schneider C. Conrad M. Bornkamm G. W. Kopf M. (2015). T Cell Lipid Peroxidation Induces Ferroptosis and Prevents Immunity to Infection. J. Exp. Med. 212, 555–568. 10.1084/jem.20140857 25824823 PMC4387287

[B69] Mavinahalli J. N. Madhumalar A. Beuerman R. W. Lane D. P. Verma C. (2010). Differences in the Transactivation Domains of P53 Family Members: a Computational Study. BMC Genomics 11, S5. 10.1186/1471-2164-11-S1-S5 PMC2822533 20158876

[B70] Meng M. Huang M. Liu C. Wang J. Ren W. Cui S. (2021). Local Anesthetic Levobupivacaine Induces Ferroptosis and Inhibits Progression by Up-Regulating P53 in Non-small Cell Lung Cancer. Aging (Albany NY) 13. 10.18632/aging.203138 34175840

[B71] Mou Y. Wang J. Wu J. He D. Zhang C. Duan C. (2019). Ferroptosis, a New Form of Cell Death: Opportunities and Challenges in Cancer. J. Hematol. Oncol. 12, 34. 10.1186/s13045-019-0720-y 30925886 PMC6441206

[B72] Oliner J. D. Pietenpol J. A. Thiagalingam S. Gyuris J. Kinzler K. W. Vogelstein B. (1993). Oncoprotein MDM2 Conceals the Activation Domain of Tumour Suppressor P53. Nature 362, 857–860. 10.1038/362857a0 8479525

[B73] Olivier M. Eeles R. Hollstein M. Khan M. A. Harris C. C. Hainaut P. (2002). The IARC TP53 Database: New Online Mutation Analysis and Recommendations to Users. Hum. Mutat. 19, 607–614. 10.1002/humu.10081 12007217

[B74] Ou Y. Wang S. J. Li D. Chu B. Gu W. (2016). Activation of SAT1 Engages Polyamine Metabolism with P53-Mediated Ferroptotic Responses. Proc. Natl. Acad. Sci. U. S. A. 113, E6806–E6812. 10.1073/pnas.1607152113 27698118 PMC5098629

[B75] Pegg A. E. (2008). Spermidine/spermine-N(1)-acetyltransferase: a Key Metabolic Regulator. Am. J. Physiol. Endocrinol. Metab. 294, E995–E1010. 10.1152/ajpendo.90217.2008 18349109

[B76] Pietsch E. C. Sykes S. M. McMahon S. B. Murphy M. E. (2008). The P53 Family and Programmed Cell Death. Oncogene 27, 6507–6521. 10.1038/onc.2008.315 18955976 PMC2657599

[B77] Rajendran R. Garva R. Ashour H. Leung T. Stratford I. Krstic-Demonacos M. (2013). Acetylation Mediated by the p300/CBP-Associated Factor Determines Cellular Energy Metabolic Pathways in Cancer. Int. J. Oncol. 42, 1961–1972. 10.3892/ijo.2013.1907 23591450

[B78] Rodriguez M. S. Desterro J. M. Lain S. Lane D. P. Hay R. T. (2000). Multiple C-Terminal Lysine Residues Target P53 for Ubiquitin-Proteasome-Mediated Degradation. Mol. Cell. Biol. 20, 8458–8467. 10.1128/MCB.20.22.8458-8467.2000 11046142 PMC102152

[B79] Ryan K. M. (2011). p53 and Autophagy in Cancer: Guardian of the Genome Meets Guardian of the Proteome. Eur. J. Cancer(Oxford, Engl. 1990) 47, 44–50. 10.1016/j.ejca.2010.10.020 21112207

[B80] Saint-Germain E. Mignacca L. Vernier M. Bobbala D. Ilangumaran S. Ferbeyre G. (2017). SOCS1 Regulates Senescence and Ferroptosis by Modulating the Expression of P53 Target Genes. Aging (Albany NY) 9, 2137–2162. 10.18632/aging.101306 29081404 PMC5680560

[B81] Shao M. Jiang Q. Shen C. Liu Z. Qiu L. (2022). Sinapine Induced Ferroptosis in Non-small Cell Lung Cancer Cells by Upregulating Transferrin/transferrin Receptor and Downregulating SLC7A11. Gene 827, 146460. 10.1016/j.gene.2022.146460 35358657

[B82] Sheng Z. Zhu J. Deng Y. N. Gao S. Liang S. (2021). SUMOylation Modification-Mediated Cell Death. Open Biol. 11, 210050. 10.1098/rsob.210050 34255975 PMC8277462

[B83] Shi X. Kachirskaia I. Yamaguchi H. West L. E. Wen H. Wang E. W. (2007). Modulation of P53 Function by SET8-Mediated Methylation at Lysine 382. Mol. Cell. 27, 636–646. 10.1016/j.molcel.2007.07.012 17707234 PMC2693209

[B84] Shieh S. Y. Ahn J. Tamai K. Taya Y. Prives C. (2000). The Human Homologs of Checkpoint Kinases Chk1 and Cds1 (Chk2) Phosphorylate P53 at Multiple DNA Damage-Inducible Sites. Genes. Dev. 14, 289–300. 10.1101/gad.14.3.289 10673501 PMC316358

[B85] Shieh S. Y. Ikeda M. Taya Y. Prives C. (1997). DNA Damage-Induced Phosphorylation of P53 Alleviates Inhibition by MDM2. Cell. 91, 325–334. 10.1016/s0092-8674(00)80416-x 9363941

[B86] Shieh S. Y. Taya Y. Prives C. (1999). DNA Damage-Inducible Phosphorylation of P53 at N-Terminal Sites Including a Novel Site, Ser20, Requires Tetramerization. EMBO J. 18, 1815–1823. 10.1093/emboj/18.7.1815 10202145 PMC1171267

[B87] Spiegelberg D. Mortensen A. C. Lundsten S. Brown C. J. Lane D. P. Nestor M. (2018). The MDM2/MDMX-P53 Antagonist PM2 Radiosensitizes Wild-type P53 Tumors. Cancer Res. 78, 5084–5093. 10.1158/0008-5472.CAN-18-0440 30026328

[B88] Stockwell B. R. Friedmann Angeli J. P. Bayir H. Bush A. I. Conrad M. Dixon S. J. (2017). Ferroptosis: A Regulated Cell Death Nexus Linking Metabolism, Redox Biology, and Disease. Cell. 171, 273–285. 10.1016/j.cell.2017.09.021 28985560 PMC5685180

[B89] Su Y. Zhao B. Zhou L. Zhang Z. Shen Y. Lv H. (2020). Ferroptosis, a Novel Pharmacological Mechanism of Anti-cancer Drugs. Cancer Lett. 483, 127–136. 10.1016/j.canlet.2020.02.015 32067993

[B90] Suzuki S. Tanaka T. Poyurovsky M. V. Nagano H. Mayama T. Ohkubo S. (2010). Phosphate-activated Glutaminase (GLS2), a P53-Inducible Regulator of Glutamine Metabolism and Reactive Oxygen Species. Proc. Natl. Acad. Sci. U. S. A. 107, 7461–7466. 10.1073/pnas.1002459107 20351271 PMC2867754

[B91] Suzuki T. Motohashi H. Yamamoto M. (2013). Toward Clinical Application of the Keap1-Nrf2 Pathway. Trends Pharmacol. Sci. 34, 340–346. 10.1016/j.tips.2013.04.005 23664668

[B92] Tarangelo A. Magtanong L. Bieging-Rolett K. T. Li Y. Ye J. Attardi L. D. (2018). p53 Suppresses Metabolic Stress-Induced Ferroptosis in Cancer Cells. Cell. Rep. 22, 569–575. 10.1016/j.celrep.2017.12.077 29346757 PMC5791910

[B93] Teufel D. P. Bycroft M. Fersht A. R. (2009). Regulation by Phosphorylation of the Relative Affinities of the N-Terminal Transactivation Domains of P53 for P300 Domains and Mdm2. Oncogene 28, 2112–2118. 10.1038/onc.2009.71 19363523 PMC2685776

[B94] Valente L. J. Gray D. H. Michalak E. M. Pinon-Hofbauer J. Egle A. Scott C. L. (2013). p53 Efficiently Suppresses Tumor Development in the Complete Absence of its Cell-Cycle Inhibitory and Proapoptotic Effectors P21, Puma, and Noxa. Cell. Rep. 3, 1339–1345. 10.1016/j.celrep.2013.04.012 23665218

[B95] Venkatesh D. O'Brien N. A. Zandkarimi F. Tong D. R. Stokes M. E. Dunn D. E. (2020). MDM2 and MDMX Promote Ferroptosis by PPARα-Mediated Lipid Remodeling. Genes. Dev. 34, 526–543. 10.1101/gad.334219.119 32079652 PMC7111265

[B96] Wade M. Li Y. C. Wahl G. M. (2013). MDM2, MDMX and P53 in Oncogenesis and Cancer Therapy. Nat. Rev. Cancer 13, 83–96. 10.1038/nrc3430 23303139 PMC4161369

[B97] Wang S. J. Li D. Ou Y. Jiang L. Chen Y. Zhao Y. (2016). Acetylation Is Crucial for P53-Mediated Ferroptosis and Tumor Suppression. Cell. Rep. 17, 366–373. 10.1016/j.celrep.2016.09.022 27705786 PMC5227654

[B98] Wei Y. Zhu Z. Hu H. Guan J. Yang B. Zhao H. (2022). Eupaformosanin Induces Apoptosis and Ferroptosis through Ubiquitination of Mutant P53 in Triple-Negative Breast Cancer. Eur. J. Pharmacol. 924, 174970. 10.1016/j.ejphar.2022.174970 35469839

[B99] Xie Y. Zhu S. Song X. Sun X. Fan Y. Liu J. (2017). The Tumor Suppressor P53 Limits Ferroptosis by Blocking DPP4 Activity. Cell. Rep. 20, 1692–1704. 10.1016/j.celrep.2017.07.055 28813679

[B100] Xu W. H. Li C. H. Jiang T. L. (2018). Ferroptosis Pathway and its Intervention Regulated by Chinese Materia Medica. Zhongguo Zhong Yao Za Zhi 43, 4019–4026. 10.19540/j.cnki.cjcmm.20180517.001 30486525

[B101] Yang T. Choi Y. Joh J. W. Cho S. K. Kim D. S. Park S. G. (2019). Phosphorylation of P53 Serine 15 Is a Predictor of Survival for Patients with Hepatocellular Carcinoma. Can. J. Gastroenterol. Hepatol. 2019, 9015453. 10.1155/2019/9015453 30881947 PMC6383407

[B102] Yang W. H. Kim J. E. Nam H. W. Ju J. W. Kim H. S. Kim Y. S. (2006). Modification of P53 with O-Linked N-Acetylglucosamine Regulates P53 Activity and Stability. Nat. Cell. Biol. 8, 1074–1083. 10.1038/ncb1470 16964247

[B103] Yuan F. Sun Q. Zhang S. Ye L. Xu Y. Deng G. (2022). The Dual Role of P62 in Ferroptosis of Glioblastoma According to P53 Status. Cell. Biosci. 12, 20. 10.1186/s13578-022-00764-z 35216629 PMC8881833

[B104] Zhang C. Lin M. Wu R. Wang X. Yang B. Levine A. J. (2011). Parkin, a P53 Target Gene, Mediates the Role of P53 in Glucose Metabolism and the Warburg Effect. Proc. Natl. Acad. Sci. U. S. A. 108, 16259–16264. 10.1073/pnas.1113884108 21930938 PMC3182683

[B105] Zhang E. Guo Q. Gao H. Xu R. Teng S. Wu Y. (2015). Metformin and Resveratrol Inhibited High Glucose-Induced Metabolic Memory of Endothelial Senescence through SIRT1/p300/p53/p21 Pathway. PLoS ONE 10, e0143814. 10.1371/journal.pone.0143814 26629991 PMC4668014

[B106] Zhang W. Gai C. Ding D. Wang F. Li W. (2018). Targeted P53 on Small-Molecules-Induced Ferroptosis in Cancers. Front. Oncol. 8, 507. 10.3389/fonc.2018.00507 30450337 PMC6224449

[B107] Zhang X. Zheng Q. Yue X. Yuan Z. Ling J. Yuan Y. (2022). ZNF498 Promotes Hepatocellular Carcinogenesis by Suppressing P53-Mediated Apoptosis and Ferroptosis via the Attenuation of P53 Ser46 Phosphorylation. J. Exp. Clin. Cancer Res. 41, 79. 10.1186/s13046-022-02288-3 35227287 PMC8883630

[B108] Zhang Y. Qian Y. Zhang J. Yan W. Jung Y. S. Chen M. (2017). Ferredoxin Reductase Is Critical for P53-dependent Tumor Suppression via Iron Regulatory Protein 2. Genes. Dev. 31, 1243–1256. 10.1101/gad.299388.117 28747430 PMC5558926

[B109] Zhao X. Sun W. Ren Y. Lu Z. (2021). Therapeutic Potential of P53 Reactivation in Cervical Cancer. Crit. Rev. Oncol. Hematol. 157, 103182. 10.1016/j.critrevonc.2020.103182 33276182

[B110] Zheng J. Conrad M. (2020). The Metabolic Underpinnings of Ferroptosis. Cell. Metab. 32, 920–937. 10.1016/j.cmet.2020.10.011 33217331

[B111] Zhou X. Zou L. Chen W. Yang T. Luo J. Wu K. (2021). Flubendazole, FDA-Approved Anthelmintic, Elicits Valid Antitumor Effects by Targeting P53 and Promoting Ferroptosis in Castration-Resistant Prostate Cancer. Pharmacol. Res. 164, 105305. 10.1016/j.phrs.2020.105305 33197601

[B112] Zhou Y. Que K. T. Zhang Z. Yi Z. J. Zhao P. X. You Y. (2018). Iron Overloaded Polarizes Macrophage to Proinflammation Phenotype through ROS/acetyl-p53 Pathway. Cancer Med. 7, 4012–4022. 10.1002/cam4.1670 29989329 PMC6089144

[B113] Zhu D. Osuka S. Zhang Z. Reichert Z. R. Yang L. Kanemura Y. (2018). Bai1 Suppresses Medulloblastoma Formation by Protecting P53 from Mdm2-Mediated Degradation. Cancer Cell. 33, 1004. 10.1016/j.ccell.2018.05.006 29894688 PMC6002773

[B114] Zhu H. Klement J. D. Lu C. Redd P. S. Yang D. Smith A. D. (2021). Asah2 Represses the P53-Hmox1 Axis to Protect Myeloid-Derived Suppressor Cells from Ferroptosis. J.I. Baltim. Md 206, 1395–1404. 10.4049/jimmunol.2000500 PMC7946776 33547170

